# Gaining insights into genomic regions associated with *Chilo partellus* resistance in teosinte-derived maize population

**DOI:** 10.3389/fgene.2025.1577830

**Published:** 2025-04-16

**Authors:** Ramandeep Kaur, Gurpreet Kaur, Navpreet, Ashmita, Jawala Jindal, Ramesh Kumar, Pardeep Kumar, Yogesh Vikal, Priti Sharma

**Affiliations:** ^1^ School of Agricultural Biotechnology, Punjab Agricultural University, Ludhiana, India; ^2^ Department of Plant Breeding and Genetics, Punjab Agricultural University, Ludhiana, India; ^3^ Indian Institute of Maize Research, Ludhiana, India

**Keywords:** Quantitative trait loci, *Chilo partellus*, genotyping by sequencing, simple sequence repeat marker, single-nucleotide polymorphism marker

## Abstract

**Introduction:**

Maize stem borer (*Chilo partellus*) is an important primary pest of the maize crop that feeds on leaves, cobs, and pith, leading to complete damage of the plant and hence lower productivity of maize. Teosinte is a wild progenitor of maize and an important source of genetic variability that possesses diverse alleles for resistance against biotic and abiotic stresses. Therefore, teosinte is a promising candidate for introducing genetic diversity into cultivated maize germplasm by domesticating its wild alleles.

**Methods:**

In this study, we investigated the genomic regions in F_6_ Teosinte derived maize mapping population (recombinant inbred lines) by crossing LM13 with Teosinte (*Zea mays sps. parviglumis*) during 2020 -2023. The F_6_ mapping population (89 lines) thus developed was subjected to genotyping by sequencing (GBS), and the polymorphic simple sequence repeat (SSR) markers were found. This population was screened against *C. partellus* {leaf injury rating (LIR) and % dead heart} during the *Kharif* seasons of 2023 and 2024 (June to September).

**Results:**

The C. partellus infestations showed significant differences among the F6 lines with respect to the measured LIR and % dead heart, where the LIR ranged from 1.7 to 7.7 in the population. The phenotypic and molecular data from the SSR and single-nucleotide polymorphism (SNP) markers were used to map the quantitative trait loci (QTLs). A total of four putative QTLs (*qLIR_4.1, qLIR_9.1, qDH_1.1*, and *qDH_2.1*) were identified on chromosomes 4, 9, 1, and 2 respectively for both the traits.

**Conclusion:**

These QTLs can be used in marker-assisted breeding to develop hybrids resistant to *C. partellus*. Based on a literature review, we believe that our study offers a pioneering report on identifying the QTLs associated with *C. partellus* resistance in maize varieties in Asia. The findings of this study are expected to be of use in the future for fine mapping, expression analyses, and marker tag development for marker-assisted selection aimed at improving maize resistance to pests.

## Introduction

Maize is an important staple crop known to provide nutritional sustenance globally and is often cultivated in tropical and temperate regions of the world. However, in recent years, various abiotic and biotic stresses have adversely affected the annual production of maize (Yadav et al., 2015). Biotic stresses are considered the major causes of yield instabilities in various maize growing areas of the world; an estimated 10% of the global maize production is lost annually owing to biotic stresses (Gong et al., 2014), with insect pests accounting for approximately 24.5% of these losses (Mugo et al., 2012). Among the biotic stressors, approximately 140 different insect species are known to affect maize to different levels of damage. Only 12 out of these 140 insect species can seriously damage maize crops at different growth stages, such as sowing and harvesting, while some other species can cause damage during storage (Siddiqui and Marwaha, 1993). Of all the pests known to infest the maize crop, only a dozen species produce serious consequences and need control measures (Siddiqui and Marwaha, 1994). Maize production is severely affected by *Chilo partellus* (lepidopteran stem borer), which is a disastrous pest as it feeds inside and outside the plant. This pest is the most abundant type and causes severe damage depending on the crop stage, where the maximal damage occurs at early growth stages (Duale and Nwanze, 1999); in severe cases, this pest can cause up to 75% yield reduction (Sharma and Gautam, 2010). *C. partellus* comprises approximately 89.5% of all stem borers effecting the maize crop (Songa et al., 2001). In Punjab state of India, a study reported a 13% reduction in grain yield during the Kharif season caused primarily by inadequate protection against pests and diseases despite the use of chemical sprays (Dhaliwal et al., 2018). Stem borers are challenging pests to manage because they hide inside the whorl and result in the formation of dead hearts when the larvae enter the plant and affect the growing tip of the central shoot (Kumar and Asino, 1993). Thus, it is nearly impossible to target pests inside the cob using biological control measures and insecticide spray solutions. Moreover, the modes of action of the recommended biological control agents are often slow and complex, making them difficult and labor-intensive to effectively control the infestation (Niazi et al., 2014). The use of pesticides to combat insects can cause detrimental effects on the environment, elimination of friendly insects, and development of resistant insect species. This can be avoided by introducing resistant hybrids/cultivars through molecular breeding. The deployment of resistant hybrids is known to be environmentally safe, cost-effective, practical, farmer friendly, and publicly satisfactory for controlling unwanted pests (Afzal et al., 2009).

Teosinte is a wild progenitor of maize (*Zea mays* ssp. *parviglumis*) and has been reported to be resistant to many biotic and abiotic stresses, including insect attacks, while possessing genetic variability for different agronomic traits (Joshi et al., 2021; Thukral et al., 2023; Varalakshmi et al., 2023). However, stress resistance can be recreated in modern maize hybrids by combining conventional breeding methods with molecular and other biotechnological approaches. Teosinte can be hybridized with elite inbred lines of maize to improve modern maize adaptability (Baltazar et al., 2005) and also incorporated into crossing programs to develop and diversify the maize germplasm. Identification of the genomic regions in maize associated with stem borer resistance through quantitative trait loci (QTLs) mapping provides an approach toward improving breeding efficiency using marker-assisted breeding techniques. There are several reports on the identification of QTLs in temperate and tropical maize cultivars for insect resistance, particularly for maize stem-borer species (Jiménez-Galindo et al., 2017). These studies clearly indicate that QTLs can be identified for resistance against stem borers, including *C. partellus* (Munyiri and Mugo, 2017).

In recent times, the genotyping by sequencing (GBS) approach has been applied to identify single-nucleotide polymorphisms (SNPs) in maize that can be utilized to develop high-density genetic linkage maps as well as identify QTLs for resistance against fusarium ear rot (Maschietto et al., 2017), gray leaf spots (Du et al., 2020), and Mediterranean corn borers (Jiménez-Galindo et al., 2017) in maize. Considering the above previous efforts, the present study aimed to identify the genetic loci that conferred resistance against stem borer and to explore the genetic relationships between the leaf injury rating (LIR), %dead hearts, and resistance in a teosinte-derived maize population.

## Materials and methods

### Generation of experimental material

Teosinte (*Z. mays* spp. *parviglumis*) and an available maize germplasm were prescreened for *C. partellus* resistance at the Department of Plant Breeding and Genetics, Punjab Agricultural University (PAU), Ludhiana, Punjab, India. The susceptible maize inbred line LM13 (JCY 3-7-1-1-1) and resistant accession teosinte (*Z. mays* spp. *parviglumis*) were used to develop the mapping population (F_6_) during the 2020–21, 2021–22, and 2022–23 growth seasons. The F_1s_ population was generated by crossing LM13 and teosinte during the kharif season of 2020 in the experimental area of the School of Agricultural Biotechnology, PAU, Ludhiana. Subsequently, the F_1s_ population was selfed to develop the F_2_ population at the Regional Maize Research and Seed Production Centre, Begusarai, Bihar, India in 2020 (off-season) (Supplementary Figure S1). An F_6_ population comprising a total of 89 progenies developed through pedigree method was used for the genotyping and phenotyping during the Kharif seasons of 2023 and 2024 at PAU, Ludhiana.

### Maintenance and multiplication of *C. partellus* culture

**FIGURE 1:**
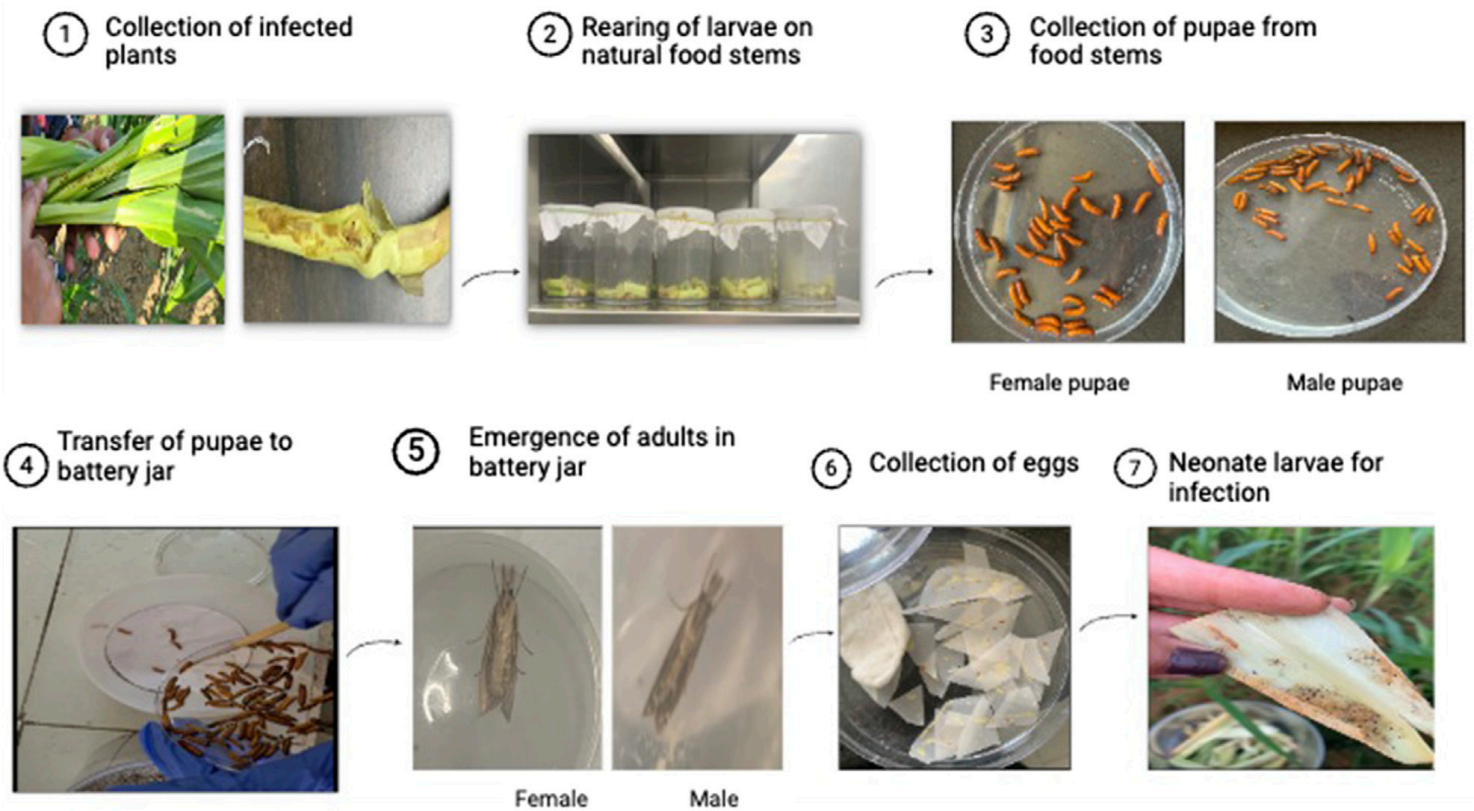
Methodology of culturing and rearing *Chilo partellus*.

A sufficient number of larvae of *C. partellus* were collected from maize crops in farmers’ fields from three different districts of Punjab, namely, Hoshiarpur, Ludhiana, and Nakodar. Larvae of different instars were collected by splitting the infected maize stalks. Further, these larvae were allowed to feed on cut pieces of soft and green maize stems (7.5 cm long) till they entered pupation. The pupae were collected from the food stems and housed in a battery jar at ambient temperature for the emergence of adults. The adults from the battery jar were transferred to oviposition jars in the proportion of one male to one female. Egg masses deposited on butter papers were collected from the oviposition jars and cut into desirable sizes. The eggs thus obtained served as a nucleus culture for the mass rearing of *C. partellus* on an artificial diet of green gram (Kanta and Sajjan, 1992) at the Maize Entomology Laboratory affiliated with the Department of Plant Breeding and Genetics, PAU, Ludhiana. From the diet jars, the adults were again collected and transferred to oviposition jars to obtain eggs; these egg masses were collected by cutting the portions of butter paper bearing the egg clusters. The egg masses were subsequently incubated for 3 d at room temperature (25°C ± 2°C) and relative humidity of 60%–70%, during which they reached the black-head stage, after which they were released into the field for uniform infestation. A diagrammatic representation of the entire methodology is given in Figure 1.

### Phenotypic evaluation

The crops were grown in a net house to prevent entry of insects from the open environment into the field area of the School of Agricultural Biotechnology, PAU, Ludhiana (Kharif 2023) and Indian Institute of Maize Research (IIMR), Ludhiana (Kharif 2024). No insecticides were used, and the recommended agronomic practices were followed to maintain a healthy crop stand. We note that natural infestation may have resulted in uneven distribution of *C. partellus* attacks within the field; therefore, it was necessary to artificially infest the maize plants with the neonate larvae of *C. partellus* to ensure that all tested plants experienced equal selection pressure.

The F_6_ population was screened under two replications during the Kharif seasons of 2023 at PAU and 2024 at IIMR in a randomized block design. Artificial infestation of each plant with neonates of *C. partellus* under field conditions was conducted after 30 d of seedling emergence (DAEs). Using a fine brush, the freshly hatched *C. partellus* larvae were picked carefully and released into the whorl of the plant. To avoid drowning of the released larvae in water retained in the whorl, the plant was gently tapped before infestation. Approximately five larvae were carefully placed in each plant avoiding injury to both the larvae and whorl. The data for LIR and %dead hearts were recorded from 10 plants in each replicate 30 d after infestation (DAI). For the LIR evaluations, a scale of 1 (healthy plant) to 9 (dead heart) was used (Sekhon et al., 1993) (Table 1). The percentage of dead hearts was calculated for each line using the following formula:

$$\frac{Number\;of\;dead\;hearts}{Total\;number\;of\;plants}\times 100$$

**TABLE 1:** Scale for leaf injury rating (LIR) as defined by Sekhon et al. (1993).

Visual Rating	Plant damage
1	Plant appears completely healthy with no visible signs of damage
2	Slight damage, such as minor pinholes, is visible on 1–2 leaves
3	Noticeable damage with pinholes or shot holes on 3–4 leaves
4	Approximately one-third of the leaves show damage (pinholes or shot holes), with possible mid-rib tunneling on 1–2 leaves
5	Approximately 50% of the leaves are damaged (pinholes, shot holes, slits, or streaks), with potential mid-rib damage
6	Plant shows various types of leaf injuries in approximately two-thirds of the total number of leaves
7	Almost all leaves show some form of damage based on various types of injuries
8	Severe damage to nearly every leaf, with stunted growth and potential for dead hearts
9	Plant exhibits dead hearts

The mean LIR and %dead heart values were calculated for each line. The predicted mean values were then subjected to analysis of variance (ANOVA) with the genotypes as the fixed effects and replication as random effects in each line across the environment using R software (R version 4.4.3). The correlation coefficient was then calculated from the average values of both parameters using R software.

### GBS and SNP identification

The DNA of the recombinant inbred line (RIL) population was extracted along with those of the parental lines using the CTAB method, and the quality and quantity of the extracted DNA was ensured using 1% agarose gel electrophoresis. For the simple sequence repeat (SSR) genotyping, a total of 230 SSR markers were used to check for polymorphism between LM13 and teosinte. The polymorphic markers were used to genotype the F_6_ population (Supplementary Table S1). Two micrograms of DNA per sample was used as the input material to construct paired-end sequencing libraries, which were then sequenced on the Illumina platform. For the GBS library preparation, a type II restriction endonuclease (*ApeKI*) was used for DNA digestion, and the digested DNAs were ligated to the adapter before constructing the 96-plex library as per the GBS protocol (Elshire et al., 2011). The unligated adapters were purified through bead-based purification. The GBS was carried out using the Illumina HiSeq2500 sequencing platform.

Quality assessments of the short raw reads were conducted using FASTQC 11.8 with the default parameters. The low-quality short reads were trimmed using Trimmomatic-0.39, and the high-quality sequences were aligned and mapped to the Zm-B73-Reference-Nam-5.0 reference genome retrieved from maizeGDB (Maize Genome Database- B73 Reference version 4.0; https://maizegdb.org/) using the BWA program. The reference genome was indexed using SAM tools prior to SNP calling and variant calling file (VCF) generation. The VCFs were filtered on the basis of the quality scores (minQ30) and total depth (minDP 4), and the indels were removed. The VCFs were converted to HapMap files using Tassel software; using the ABH-plugin in the Tassel pipeline, the filtered SNPs were converted to ABH format, where “A” represents the donor allele, “B” represents the recipient allele, and “H” represents the heterozygous allele. Finally, only the polymorphic SNPs were retained for construction of the linkage map.

### Linkage map construction and QTL mapping

To construct the linkage map, the filtered SNPs and SSR markers were used, where markers missing in one or both parents were excluded. A chi-squared test was conducted, and the non-significant markers were selected. These markers were grouped on the basis of the logarithm of odd scores (LOD) threshold score of 3.0 as well as recombination frequency of 0.3. Then, the markers were ordered using the K-optimality algorithm by recombination using the random nearest neighbor (NN) count route (10 iterations). Rippling was then performed to refine the marker order on each chromosome using the sum of adjacent recombination fractions (SARFs) algorithm with its default window size. The final output was used to generate the linkage map, and the most likely marker order for each linkage group was reconfirmed and finalized using ICI mapping software (Meng et al., 2015). The genotypic data with genetic distances between the markers and average phenotypic data for the LIR and %dead hearts were used to map the QTLs; the biparental populations (BIPs) functionality of the ICI mapping software was used to study the associations of the markers with LIR and %dead hearts. The inclusive composite interval mapping (ICIM) method with additive effects (i.e., ICIM-ADD) was used to map the QTLs at the LOD threshold of 2.5. The effects of the QTLs were estimated based on the LOD, additive effects of the identified loci, and percentage of phenotypic variation explained (PVE%).

### Identification of candidate genes

The QTL regions were mined for the presence of genes conferring resistance against insect pests. An *in silico* strategy was used to mine candidate genes within the identified QTL regions flanked by the linked markers. Here, the sequences of the two markers flanking the target QTL regions were located on the chromosome region of the sequence map based on the genome positions using BLAST from the assembled maize physical map obtained from maizeGDB. Then, the genomic sequence within the two target markers was retrieved from the database, followed by prediction of genes associated with the target QTL regions using maizeGDB. Furthermore, the functions related to the genes were identified from Pfam, NCBI, and maizeGDB.

## Results

### Correlation and phenotypic evaluation

**FIGURE 2:**
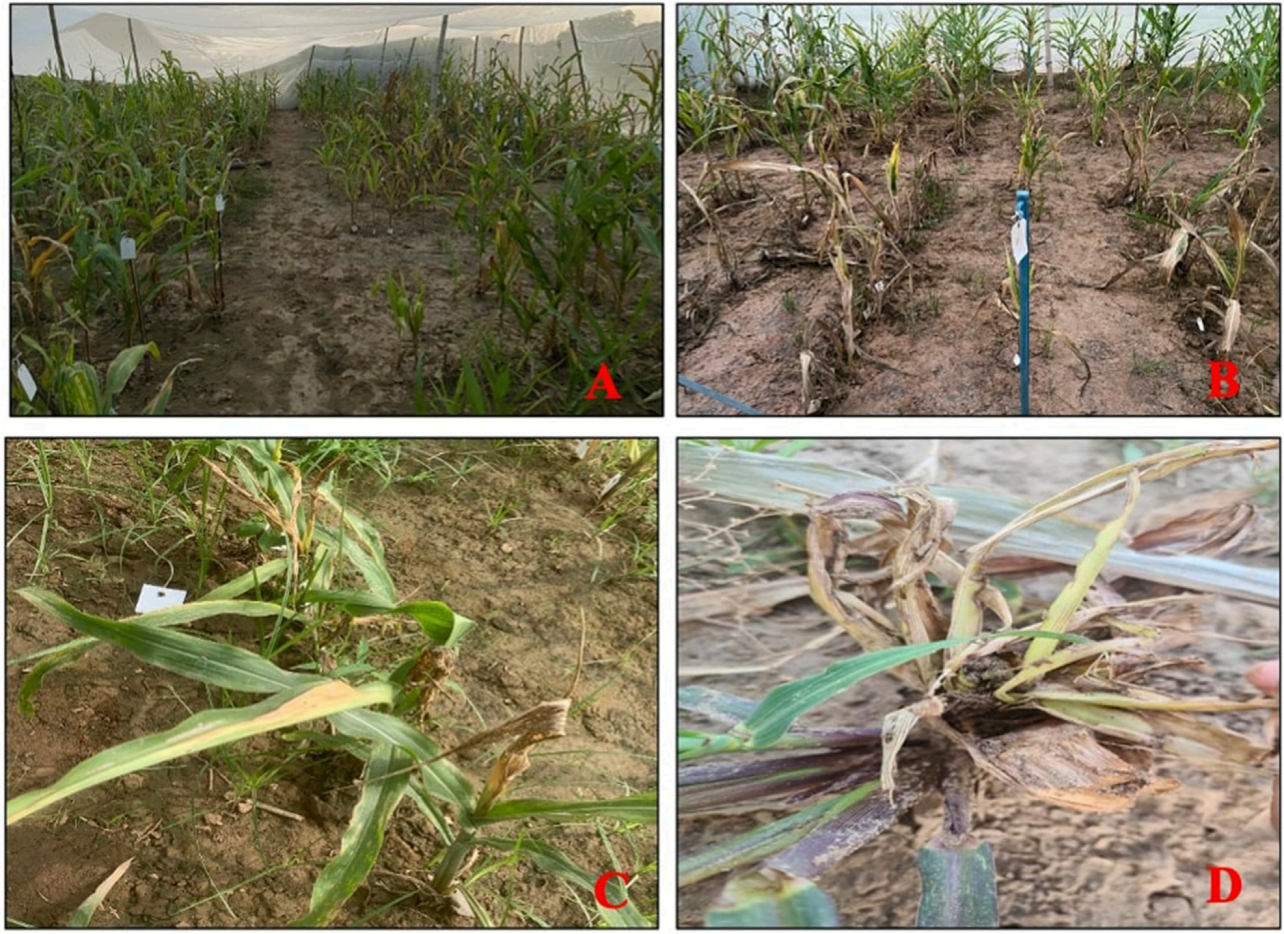
**(A, B)** Screening of the F_6_ population against *Chilo partellus* in the field; **(C, D)** dead heart formation.

**FIGURE 3:**
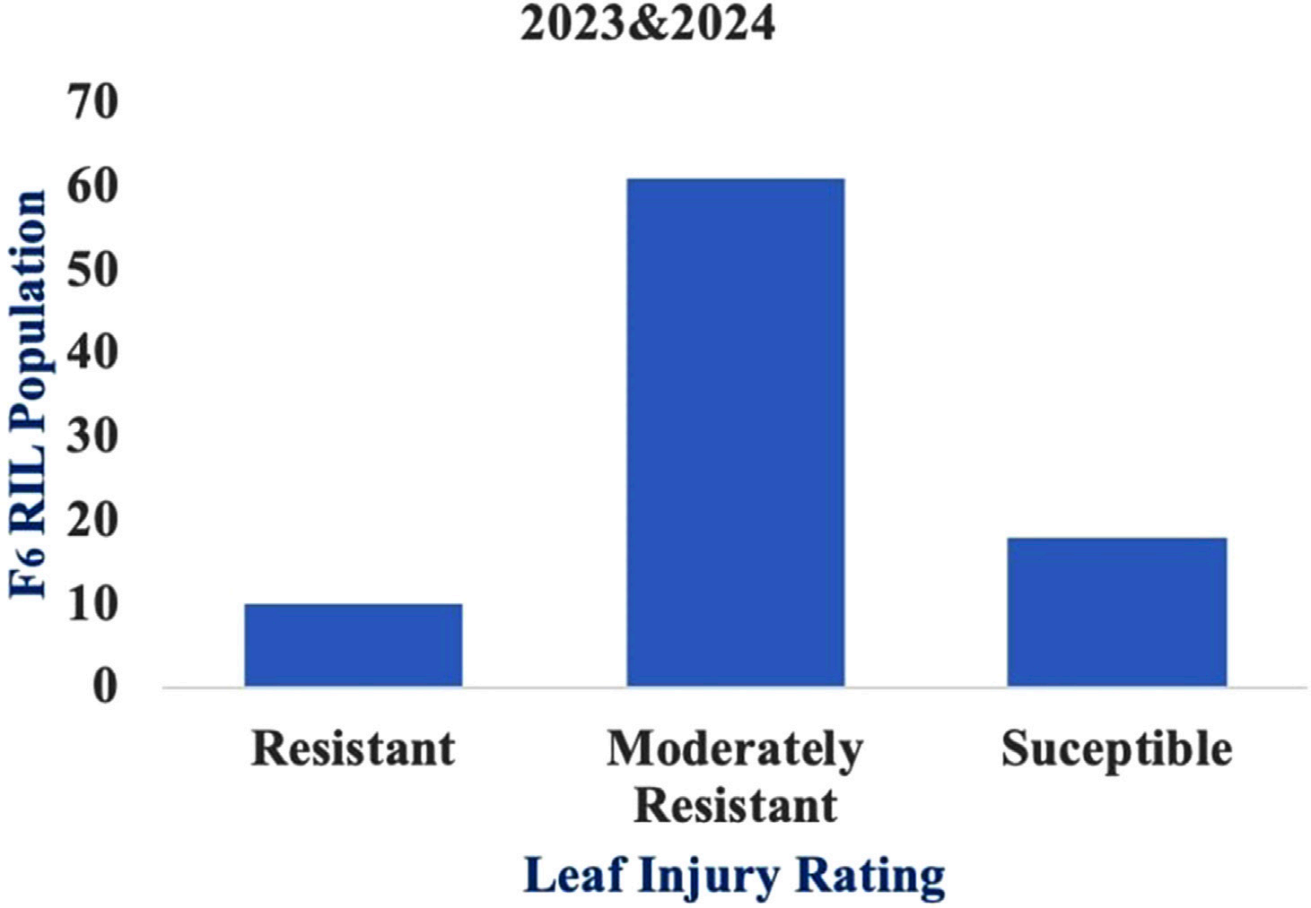
Frequency distribution of the recombinant inbred lines (RILs) according to leaf injury ratings (LIRs).

**FIGURE 4:**
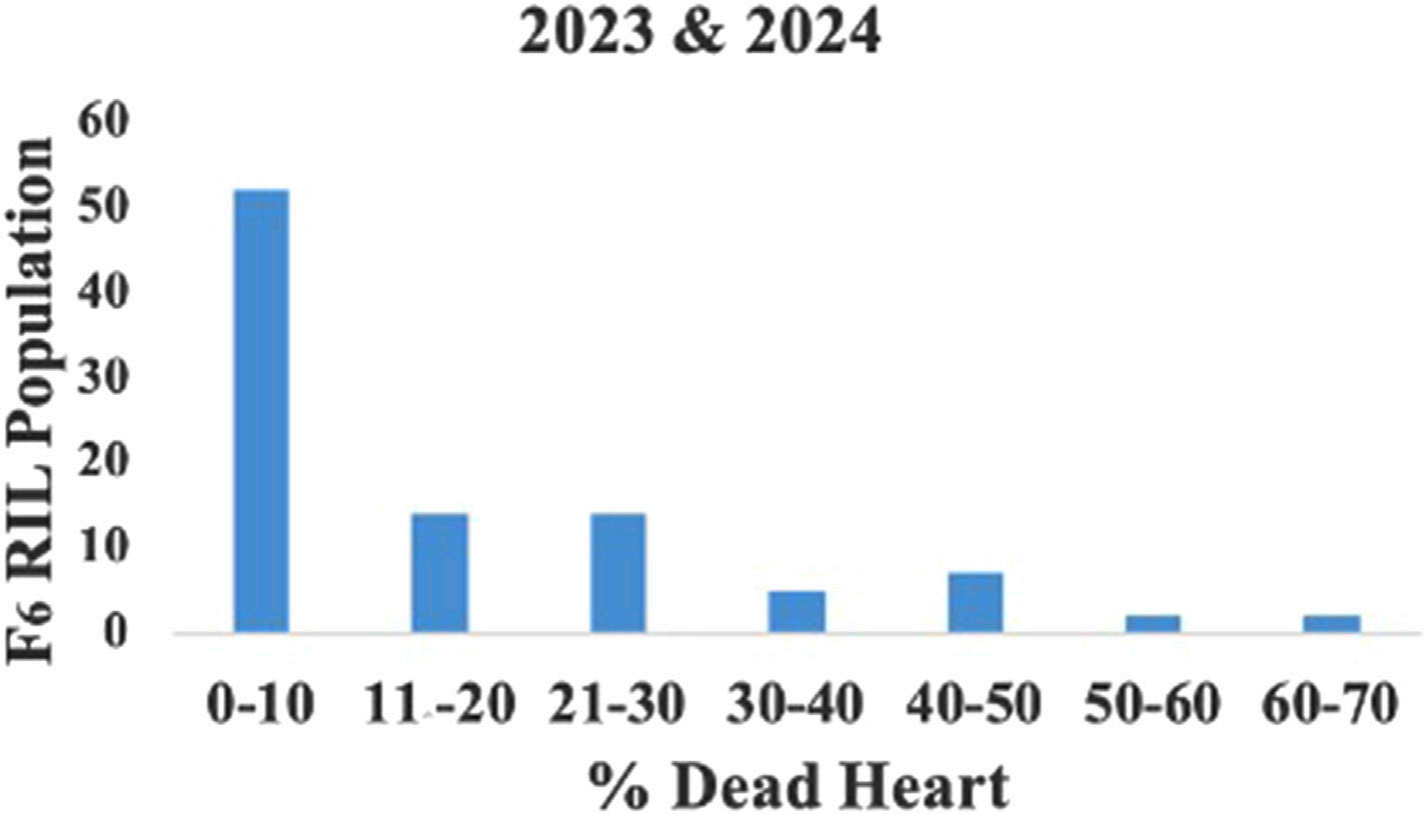
Frequency distribution of the RILs according to % dead hearts.

The mean LIR and %dead hearts for each RIL and parental lines of the F_6_ population were calculated from data collected during 2023 and 2024 from both study locations. As both study locations remained the same over the years, the data were pooled to compute the average for further analyses. The screening results showed that the genotypes were significantly different in response to *C. partellus* attack (Figure 2) and that the mean LIR was in the range of 1.7–7.7 during Kharif 2023–2024. The %dead hearts values ranged from 0% to 50%. The maximum mean values of the LIR and %dead hearts were noted for RIL 57 (7.7 and 50.1%, respectively) while the minimum values were observed for RIL 54 (1.7 and 0.0%, respectively) (Table 2). The susceptible parental line LM13 showed a mean LIR of 6.11, whereas the resistant parent teosinte showed a mean LIR of 2.25. The RIL distributions according to the LIR scale and %dead hearts are shown in Figures 3, 4. The mean LIR was evaluated through ANOVA and correlation analysis. The combined ANOVA data were used to test for statistical significance among the genotypes and replications for LIR and %dead hearts. The genotypes showed significant differences for LIR, while the effects of replications were non-significant for LIR (Table 3). The LIR was also significantly influenced by genotype × environment interaction (*p* ≤ 0.05). For the %dead hearts, both replications (*p* ≤ 0.01) and genotypes (*p* ≤ 0.001) showed significant differences. A positive and significant correlation coefficient of 0.484 was observed between the LIR and %dead hearts (Table 4).

**TABLE 2:** Mean, range, and standard deviation for LIR and %dead hearts.

Kharif 2023–24	Mean LIR	% Dead heart
LM13	6.11	42.90
Teosinte	2.25	0
RIL population	4.9	9.5
Range	1.7–7.7	0–50
Standard deviation	1.45	12.07

**TABLE 3:** Analysis of variance for LIR and %dead hearts.

Parameter	Df	LIR	% Dead heart
Replication	1	2.341	879.9**
Environment	1	1.448	225.8
Genotype	88	7.970***	573.8***
Genotype × Environment	88	0.137*	37.9
F value	-	10.941	6.746
Coefficient of variance	-	30.20	124.4

^*^ *p* ≤ 0.05, **p ≤ 0.01, ***p ≤ 0.001 probability levels; Df = number of degrees of freedom; LIR, leaf injury rating.

**TABLE 4:** Pearson’s correlation for LIR and %dead hearts.

	LIR	% Dead heart
LIR	1	0.484*
% Dead hearts	0.484*	1

^*^ *p* ≤ 0.001 probability level; LIR, leaf injury rating.

### Genotyping of the mapping population

The raw VCFs comprised 2,774,759 sites; after removing 100% of the missing data and retaining variants that had a minimum mean depth of 4, only 1,679,005 sites remained. These sites were further filtered according to the parental lines, following which a total of 7,049 sites remained. Additionally, 43 polymorphic SSR markers were genotyped on 89 RILs along with the parental lines. The SNPs combined with SSRs were filtered according to the chi-squared test, and a total of 2,147 markers were used to construct the linkage and QTL maps.

### QTL mapping

**FIGURE 5:**
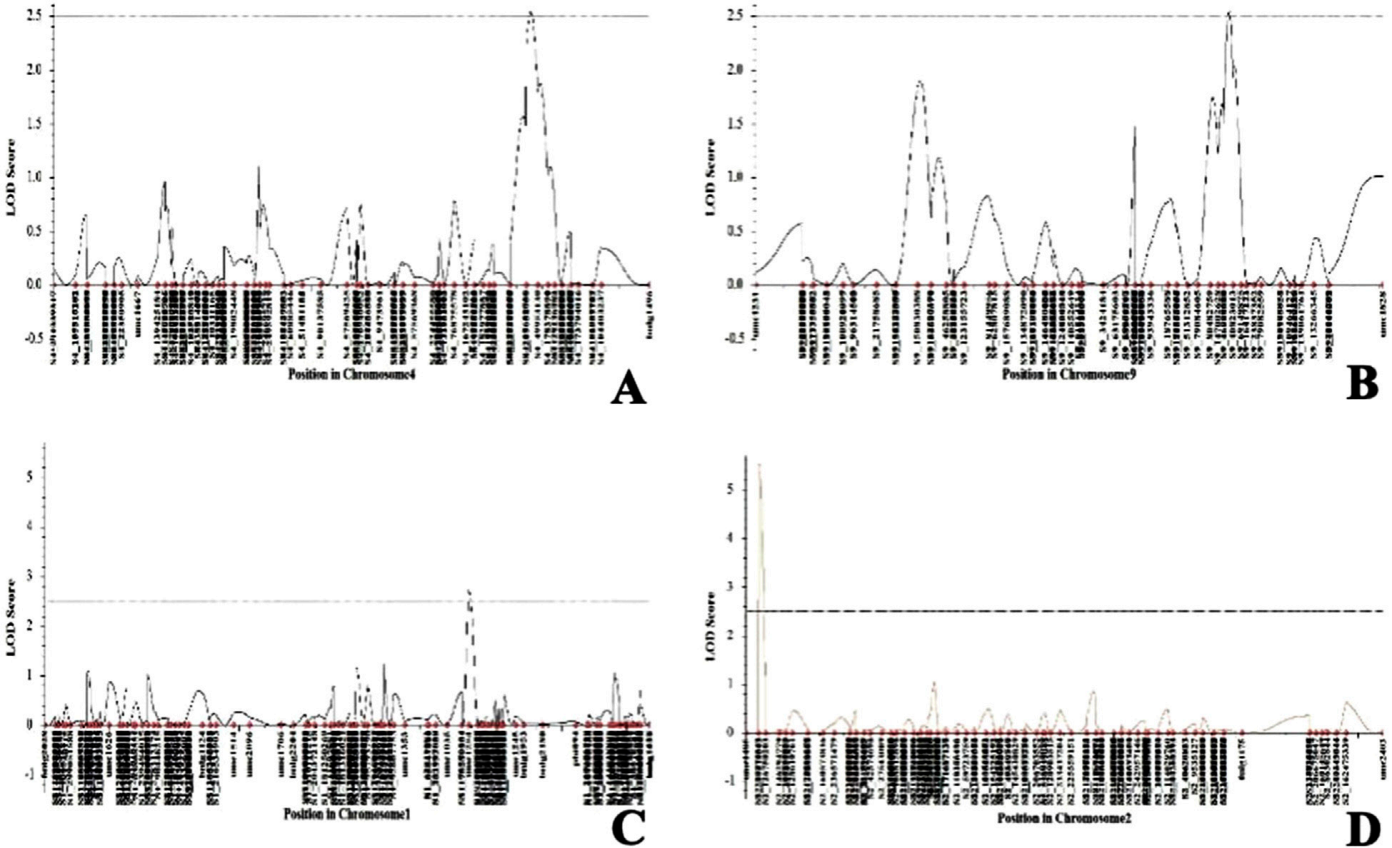
Quantitative trait loci (QTL) likelihood plots of **(A)** chromosome 4 showing the putative locus for LIR, **(B)** chromosome 9 showing the putative locus for LIR, **(C)** chromosome 1 showing the putative locus for %dead hearts, and **(D)** chromosome 2 showing the putative locus for %dead hearts.

The genotypic data of the 89 RILs with SSR markers and SNPs were analyzed using ICI mapping software to construct the genetic linkage map based on an LOD of 2.5 and recombination fraction of 0.3. The SSR and SNP markers were grouped into linkage groups. The linkage groups of each of the chromosomes spanning the number of markers and genetic distance of each chromosome are presented in Table 5 (Supplementary Figure S2). The complete genetic map had a size of 5471.51 cM. The genetic distances of all markers were calculated from the linkage maps and used to map the QTLs. Both the genotypic data of SSR and SNP markers as well as the pooled phenotypic data of the F_6_ populations of Kharif 2023 and Kharif 2024 were used in the QTL mapping for *C. partellus* resistance. The first QTL (*qLIR_9.1*) based on the mean LIR (2023–24) was identified with an LOD of 2.97 and phenotypic variance of 13.05% on chromosome 9 (Figure 5). The second QTL (*qLIR_4.1*) based on the mean LIR (2023–24) was observed on chromosome 4, which explained the phenotypic variance of 12.85% (Figure 5). The third QTL (*qDH_2.1*) based on %dead hearts (2023–24) was identified between marker intervals S2_89773662 and S2_126750203 with an LOD of 5.52 that explained 21.35% of the phenotypic variance on chromosome 2 (Figure 5). Similarly, a fourth QTL (*qDH_1.1*) based on %dead hearts (2023–24) was identified between marker intervals umc1254 and S1_216786898 with an LOD of 2.73 that explained the 9.00% phenotypic variance on chromosome 1 (Figure 5) (Table 6).

**TABLE 5:** Linkage groups spanning the number of markers and genetic distance.

Linkage group	Number of markers	Map length (cM)
Chromosome1	315	936.37
Chromosome2	274	520.29
Chromosome3	243	526.65
Chromosome4	235	463.48
Chromosome5	200	517.32
Chromosome6	201	548.30
Chromosome7	197	481.53
Chromosome8	200	434.42
Chromosome9	126	462.48
Chromosome10	156	580.73
**Total map length: 5,471.51 cM**		
**Total number of markers: 2,147**		

**TABLE 6:** Marker intervals showing the associations of putative QTLs with LIR and % Dead hearts analyzed by composite interval mapping.

Trait Name	QTL name	Chromosome	Left to right markers	LOD	PVE (%)	Add
LIR 2023–24	*qLIR_4.1*	4	S4_213631006 to S4_49953130	2.53	12.85	−0.622
LIR 2023–24	*qLIR_9.1*	9	S9_16990082 to S9_40823013	2.54	13.05	0.637
% Dead hearts 2023–24	*qDH_1.1*	1	umc1254 to S1_216786898	2.73	9.00	−3.611
% Dead hearts 2023–24	*qDH_2.1*	2	S2_89773662 to S2_126750203	5.52	21.35	−5.551

LOD, logarithm of odd scores; PVE (%), phenotypic variance; Add, additive effect.

### Candidate gene identification

Exploration of the genes in the QTL regions based on LIR (*qLIR_9.1*) (S9_16990082 to S9_40823013) and %dead hearts (*qDH_2.1*) (S2_89773662 to S2_126750203) was carried out on chromosome 9 and 2, respectively, using the genetic distance information along with the start and end positions of the markers from the map files. The gene sequences were obtained from maizeGDB and searched for domains using Pfam. The genes were then annotated according to the function provided in maizeGDB. A total of 162 genes were fetched for *qLIR_9.1*; a detailed analysis of the *qLIR_9.1* region revealed a total of 32 genes that could be involved in biotic stresses, where most played significant roles in insect resistance (Table 7), and the remaining genes within the QTL region are shown in Supplementary Table S2. In the case of *qDH_2.*1, approximately 100 protein coding genes were characterized, where several of the genes were found to be potentially linked with biotic stress resistance (Table 7); the remaining genes are listed in Supplementary Table S3. Maize predominantly encodes enzymes like superoxide dismutase, mitogen-activated protein kinases, and aspartic proteases that help the plant defend itself against insects. These are a part of the plant immune system that protects the plant from environmental factors. The presence of cysteine synthase leads to higher cysteine levels, which is crucial for maize insect resistance 1-cysteine protease (Mir1-CP) that enables the plant to produce more protective proteins to combat insect feeding.

**TABLE 7:** Descriptions and functions of genes in the identified QTLs.

Gene id	Description
***qLIR_9.1***	
LOC100277313	Zinc-finger-like superfamily protein and nudix hydrolase homolog
wx1	Waxy
LOC100274012	Fe superoxide dismutase
LOC103639865	SNF1-related protein kinase
LOC100281381	S-adenosyl-L-methionine-dependent methyltransferases superfamily
LOC100192103	Sulfotransferase
LOC109942208	Proline-rich receptor-like protein
LOC103638154	Disease resistance
LOC109942380	Acyl activating enzyme
LOC103639885	Phosphatidylinositol transferase
LOC100382424	Acyl coenzyme oxidase
LOC100282658	Nudix hydrolase homolog
nfa101	Nucleosome chromatin assembly factor
mpk2	MAP kinase
LOC103639802	L-type receptor
LOC103638261	LRR receptor kinase
LOC103638112	Kinesin-like protein
LOC103638125	Inositol-3-phosphate synthase
LOC103638205	Helicase protein
LOC109942181	G-type lectin receptor kinase
LOC103638191	Glycine tRNA ligase
ftr1	Ferredoxin–thioredoxin
LOC103638043	Ethylene-responsive transcription factor
pco095664	DUF1995 domain
LOC100274222	DNA repair protein
LOC103638079	Cysteine synthase
LOC100280120	Aspartyl protease
asn1	Asparagine synthetase
LOC103638134	Arginine decarboxylase
LOC100282603	Anthocyanidin-O-glucosyltransferase
LOC100191469	Acyl coenzyme oxidase
LOC100285906	Beta galactosyltransferase
***qDH_2.1***	
GRMZM2G329222	ABC transporter family member
GRMZM2G125704	Calcium-dependent protein kinase substrate protein
GRMZM2G125728	Clathrin interactor EPSIN 1
GRMZM2G166281	Cysteine protease inhibitor complex
GRMZM2G106056	Double-strand strand break repair protein MRE11
GRMZM2G165007	F-box repeat protein
GRMZM2G072518	Leucine-rich repeat
GRMZM2G005107	MADS transcription factor
GRMZM2G017654	MAP3K K epsilon protein
GRMZM2G053909	Putative zinc finger domain superfamily protein
GRMZM2G106413	Wound-induced protein
GRMZM2G106393	Wound-induced protein
GRMZM2G084819	Zinc finger C3HC4 type family protein
GRMZM2G066169	Zinc-finger-domain-containing protein

## Discussion

### Phenotypic data evaluation

Insect pest attacks constitute a significant type of biotic stress among the various stresses limiting maize production. Among these, stem borers pose substantial threats to global maize yields. The maize germplasm and its wild progenitor (teosinte) serve as a reservoir of genes with potential for maize improvement and further exploration (Pressoir and Berthaud, 2004). Teosinte has been shown to be resistant to various pests, including *C. partellus,* as reported by Niazi et al. (2014). In the present study, we attempted to map *C. partellus* resistance in maize; accordingly, 89 different F_6_ progenies were developed by crossing a susceptible parent LM13 (JCY 3-7-1-1-1) with the resistant parent teosinte (*Z. mays* spp. *parviglumis*). While developing a mapping population, it is desirable to have large number of individuals (>150); however, we analyzed a relatively small population size (89) while establishing marker trait associations. The main reason for this was that during the filial generation, the susceptible lines were lost as the insect caused dead hearts leading to loss of the susceptible progenies during artificial phenotyping for the trait. Similar loss of lines were earlier reported by Reddy et al. (1989), who documented significant stalk damage of up to 80% in maize, and by Kumar et al. (2017) who reported 39.47% damage; however, these studies were not related to trait mapping.

Moreover, the lower availability of pollen per plant in teosinte and non-synchronization of flowering with the maize inbred line makes it a little difficult to achieve cross-hybridization (Cruz-Cárdenas et al., 2019). Understanding the responses of the genotypes to *C. partellus* could provide valuable insights for the selection of maize lines. Nonetheless, LIR assessment has been used as the main criterion for evaluating the genotypes against stem borers over the past four decades. Since variations between the genotypes are larger in proportion to LIR, artificial infestation is the most effective method of finding resistant genotypes compared to natural infestation. In the current investigation, the genotypes showed considerable variation in LIR owing to damage caused by *C. partellus* infestation. Correspondingly, the LIR and %dead hearts values were used to evaluate the maize cultivars for resistance against *C. partellus* (Vishvendra et al., 2017). Artificial infestation of *C. partellus* was used to screen 110 maize inbred lines, out of which 19 inbred lines were resistant, 29 inbred lines exhibited moderately susceptible reactions, 62 inbred lines were categorized as susceptible, and the remaining 13 inbred lines were highly susceptible based on the LIR scale (Sekhar et al., 2016). Sarup (1983) screened 188 F_2_ progenies (LM13 × teosinte) for *C. partellus* resistance; based on the leaf injury scale, 56 plants were found to be resistant, 112 plants were moderately resistant, and 20 plants were susceptible. In our study, out of the 89 RILs, 10 lines were resistant, 62 lines were moderately resistant, and 17 lines were susceptible based on the LIR scale. We observed that the genotypes were significantly different in response to *C. partellus* attacks and that the mean LIRs were in the range of 1.7–7.5 in the F_6_ population. Similarly, Cholla et al. (2018) evaluated approximately 30 maize genotypes against *C. partellus* resistance using artificial infestation and recorded LIRs in the range of 2.16–8.74; they observed significant and positive correlations between the LIRs, dead hearts, and tunnel lengths. The leaf damage and dead hearts were also found to be positively correlated in the sorghum population studied by Muturi et al. (2021). Similarly, the present study shows a significant and positive correlation (0.484) between the %dead hearts and LIR, strongly suggesting that these two damage criteria are crucial for identifying resistance.

### Mapping of the QTLs

The QTLs linked to resistance against two significant stem borer pests in maize production in Kenya, namely, *Busseola fusca* and *C. partellus*, were identified in an earlier study. A total of 203 F_2:3_ individuals were developed by crossing CML442 (susceptible) with CKSBL10026 (resistant) varieties, and the QTLs were mapped via 152 SNPs (Munyiri and Mugo, 2017). Data on leaf damage, stem tunneling lengths, and stem borer exit holes were collected as potential characteristics of stem borer damage. One QTL for reduced stem tunneling was found on chromosome 4 in *B. fusca*, while two QTLs were found for reduced stem tunneling and reduced stem borer exit holes on chromosomes 4 and 5, respectively, in *C. partellus*. The findings of the present study are in correspondence with these findings. The present study revealed QTLs for LIR on chromosome 4 and 9 in the F_6_ population (LM13 × teosinte spp. *parviglumis*). Several findings have been published on linkage and QTL mapping for borer resistance traits in maize (Jiménez-Galindo et al., 2017; Womack et al., 2018). Approximately 12 significant QTLs were identified on chromosomes 1, 2, 4, 5, 8, 9, and 10 for resistance against the mediterranean maize borer and agronomic traits (Jiménez-Galindo et al., 2017). Five common intervals were identified on chromosomes 3, 4, and 5 linked to fusarium ear rot resistance by comparison of the linkage and association mappings; of these, four intervals were confirmed in various near-isogenic line (NIL) populations (Wu et al., 2020). A QTL for insect leaf-feeding was found on chromosome 9 under different genetic backgrounds in several studies, including the present work (Groh et al., 1998b; Khairallah et al., 1998; Willcox et al., 2002; Brooks et al., 2005, 2007; Womack et al., 2018). This large-effect QTL is anticipated to be reliably functional in novel genetic environments after validation and could be targeted for marker-assisted selection (MAS) introgression breeding. A framework for pyramiding multiple QTLs for resistance to similar leaf-feeding insects, such as the European maize borer (Jampatong et al., 2002), Asian corn borer (Xia et al., 2010), southwestern corn borer (Brooks et al., 2007), and sugarcane borer (Bohn et al., 1996, 1997; Groh et al., 1998a), has been identified in various studies for maize insect resistance. Thus, breeders may have more opportunities to use MAS to increase maize resistance to leaf-feeding insects in the common region. The significant impacts of the QTLs on LIR and %dead hearts offer a novel genetic source of resistance in maize.

The QTLs *qLIR_9.1* and *qDH_9.1* for LIR and %dead hearts explain the phenotypic variances of approximately 13.05% and 12.05% with LOD values of 2.54 and 2.63, respectively, on chromosome 9. These LOD values of the QTLs for LIR and %dead hearts were below 3.0 (at *p <* 0.05), which is likely attributable to the low heritability of traits and variations in the phenotype being studied. Moreover, given the small population size, the probability of localization of strong QTLs is typically low unless the QTLs show significant proportions of genetic variance (Munyiri and Mugo, 2017). Thus, the low heritability for trait, i.e., stem borer resistance, showing polygenic nature should not be seen as a barrier to maize breeding programs (Bohn et al., 2000; Garcia-Lara et al., 2009).

### *In silico* gene identification

Plants evolve rapid response strategies to unfavorable conditions, and these responses often involve interconnected networks controlled by signal cascades at the molecular level. In addition to QTL analysis in this study, a total of 32 genes (*qLIR_9.1*) were identified for biotic stress resistance and annotated from maizeGDB and Pfam. The gene *LOC103638261* identified *in silco* consists of leucine-rich repeat (LRR) domains, which have been found to play important roles in biotic stress resistance. In a study by Hayford et al. (2024), two LRR-RLK genes (*Zm00001eb293660* and *Zm00001eb153630*) were identified as hub genes in the coexpression network of a meta-analysis study in maize during multiple pathogen stresses; these were also identified in a list of biotic regulated differentially expressed genes (DEGs), supporting their critical roles in biotic stress responses. Another gene (*LOC103639885*) having phosphotransferase activity was identified using Pfam in our findings. Studies have reported that protein kinases or phosphatases can upregulate or downregulate specific transcription factors and that these transcription factors bind to the *cis*-elements of stress-related genes to enhance or suppress their transcription (Bailo et al., 2019). Such genes have been found to be involved in biotic resistance in maize. The proline receptor protein, nudix hydrolase, acyl activating enzyme, acyl oxidase, S-adenosyl-methionine-dependent methyltransferase, asparagine synthase, uracil-DNA glycosylase, tRNA methyltransferase catalytic subunit, and phosphatidylinositol transferase genes were also found in the present study. These genes are shown to be involved in redox balance regulation, oxidative stress responses, and substrate level modulations to preserve physiological homeostasis (Ge and Xia, 2008; Soonil et al., 2009; Lai et al., 2021; Pinneh et al., 2019).

The present study reports genes with zinc finger protein and DUF1995 domains. It has been demonstrated that plant stress responses are mediated by the genes for several transcription factors, such as members of the zinc finger protein, DUF 1995 domain, MAP kinase, and LRR-type receptor kinase families (Gourcilleau et al., 2011; Zheng et al., 2021; Soltabayeva et al., 2022; Zhao et al., 2021). Glossy phenotypes are typically resistant to insect damage and exhibit less epicuticular wax deposition. Wax biosynthesis is mediated by the *wx1* genes. Our analysis revealed that the *wx1* gene in the QTL region could be involved in wax deposition and may confer resistance against insect pests. Another study reported that the glossy 15 (*gl15*) gene on chromosome 9 could be a potential source of resistance against the fall army worm, southwestern corn borer, and European corn borer (Jampotang et al., 2002). Insect resistance in maize involves complex defense mechanisms, including signaling pathways, metabolic adjustments, and secondary metabolite production. Among the candidate genes, mitogen-activated protein kinase 2 (*mpk2*) plays a pivotal role in regulating the jasmonic acid (JA) pathway, which is crucial for activating defense responses to herbivore feeding, including the production of protease inhibitors that deter insect feeding (Pieterse et al., 2012). Additionally, asparagine synthetase 1 (*asn1*) involved in nitrogen metabolism is known to enhance plant resilience by modulating nitrogen allocation, supporting protein synthesis, and promoting the production of defense-related proteins that strengthen plant responses to insect herbivory (López-Bucio et al., 2003). The *LOC103638205* gene may be further involved in signal transduction that activates secondary metabolite production, such as phenolics or alkaloids that act as toxins or deterrents to herbivores. Together, these genes contribute to the ability of maize to resist insect pests through a combination of metabolic regulation, hormone signaling, and production of defensive compounds.

Through comprehensive analysis, a total of 100 genes were identified within the region (*qDH_2.1*); upon further investigation, a subset of these genes was found to be significantly involved in biotic stress responses. The wound-induced proteins identified in this study are involved in signaling pathways that help the plant respond to stress. For example, JA is a key plant hormone that plays a crucial role in regulating wound responses. These proteins can be activated by JA and in turn trigger the production of other defensive proteins and metabolites (Leon et al., 2001). Additionally, ABC transporters are involved in modulating the levels and transport of hormones like JA and salicylic acid that are essential for regulating defense responses in plants (Do et al., 2018). Calcium-dependent protein kinases are also significant in plants as they participate in the signaling of stress hormones and activation of plant defense mechanisms in response to both biotic and abiotic stresses (Kiselev et al., 2025). F-box proteins play critical roles in insect resistance by regulating the ubiquitin-proteasome pathway that controls the key proteins involved in resistance mechanisms (Zhang et al., 2019). Moreover, MADS-box transcription factors are involved in regulating plant responses to biotic stresses, thereby influencing the expressions of defense-related genes (Kundu et al., 2021). Finally, cysteine protease inhibitors are known to be effective against certain insect species, further suggesting the roles of these inhibitors in plant resistance against biotic stresses (Haq et al., 2004). The identification of these genes provides valuable insights into the genetic basis of plant resistance as well as enables potential applications in crop improvement and stress resistance.

## Conclusion

A teosinte-derived maize population was developed in this study. Based on phenotypic data, RIL numbers 1, 12, 20, 54, 58, 59, 73, 74, and 75 were found to be resistant to *C. partellus* infestation*.* The QTL (*qLIR_9.1*) based on LIR was found between S9_16990082 and S9_40823013, with an LOD of 2.54 and phenotypic variance of 13.05%. From the QTL analysis, we concluded that LIR is controlled by polygenes and is complex in nature. The genes within the flanking markers were identified and annotated for use in further expression analyses. Furthermore, the identified QTLs can be transferred to other elite inbred lines, including parental lines of popular high-yield hybrids. To the best of our knowledge, the present study is a pilot effort on mapping *C. partellus* resistance in maize in India. The transfer of the identified QTLs is expected to play a significant role in developing germplasms tolerant to *C. partellus* infestation, which in turn is expected to increase maize production.

## Data Availability

The datasets presented in this study can be found in online repositories. The name of the repository and accession numbers can be found below: https://www.ncbi.nlm.nih.gov/, PRJNA1219800.
