# Supplementary material for: Gaining insights into genomic regions associated with Chilo partellus resistance in teosinte-derived maize population
Source: Front Genet. 2025 Apr 16;16:1577830. doi: 10.3389/fgene.2025.1577830 (PMC12041033; doi:10.3389/fgene.2025.1577830)
Supplement: Supplementary file 1 [file Supplementaryfile1.docx]

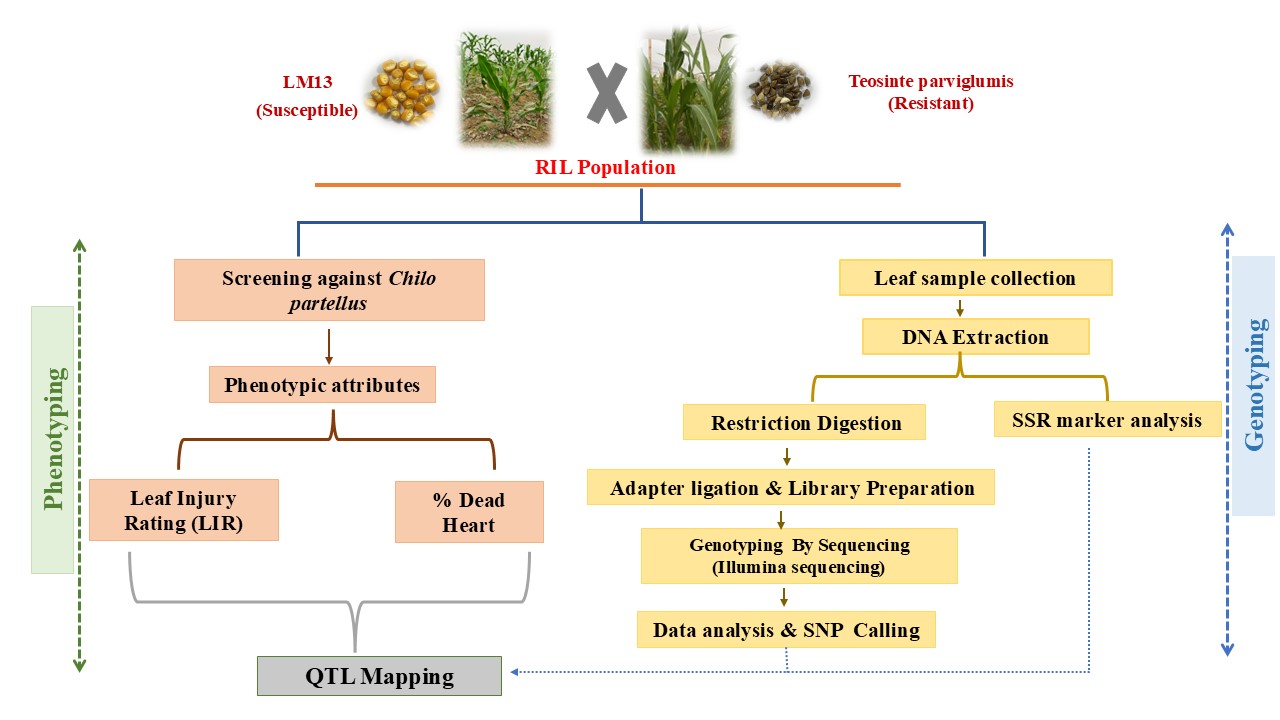


**Supplementary Figure 1.: Integration of GBS derived SNP, SSR markers and phenotyping for mapping of QTLs for *Chilo partellus* resistance in *Z. mays***


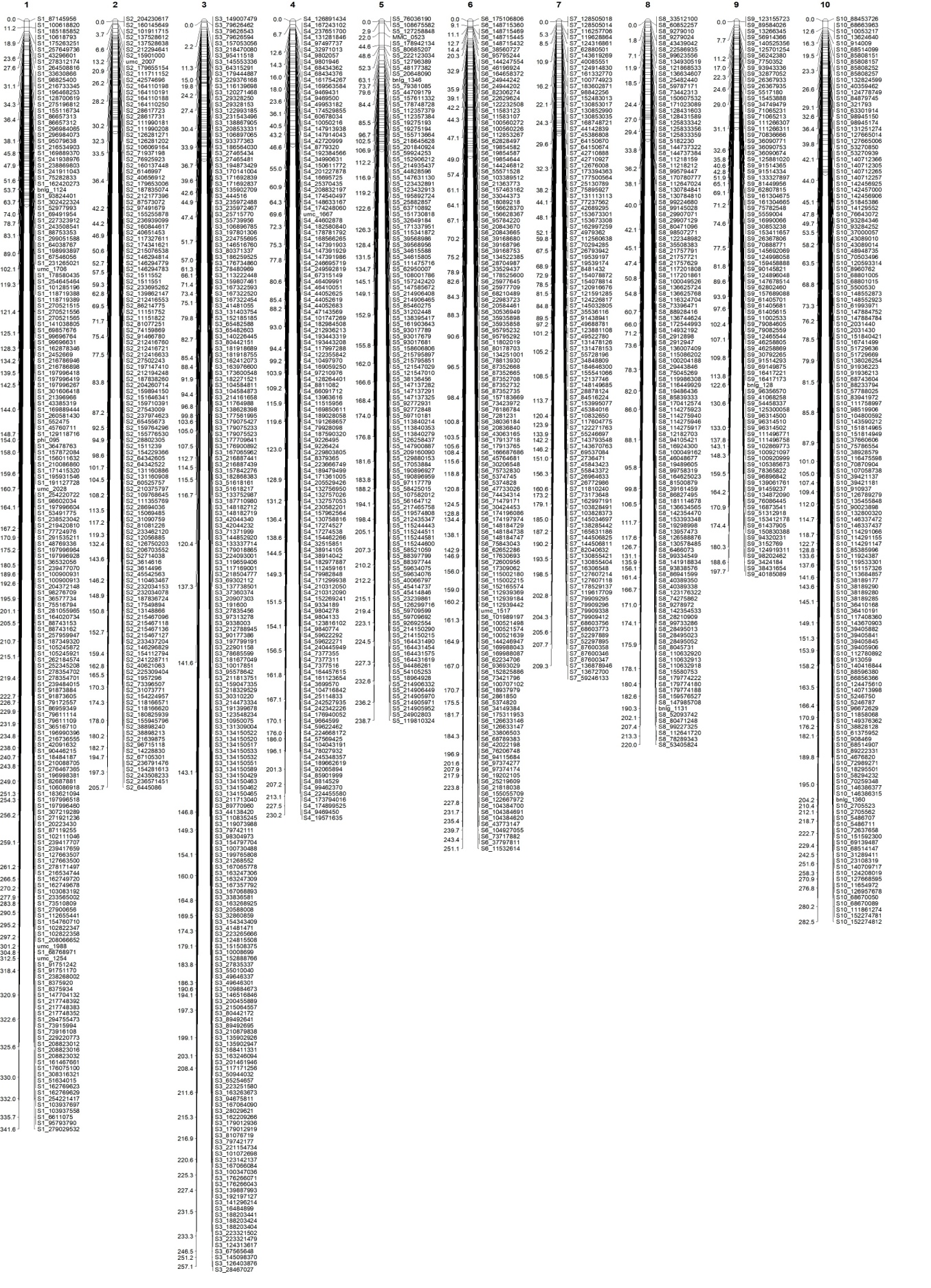


**Supplementary Figure 2.: Linkage groups developed from Teosinte derived RIL population**
