# Supplementary material for: Gaining insights into genomic regions associated with Chilo partellus resistance in teosinte-derived maize population
Source: Front Genet. 2025 Apr 16;16:1577830. doi: 10.3389/fgene.2025.1577830 (PMC12041033; doi:10.3389/fgene.2025.1577830)
Supplement: Supplementary file 2 [file Supplementaryfile2.docx]

**Supplementary Table1: List of polymorphic markers**

| **S. No.** | **SSR Marker name** | **Chromosome no.** |
| --- | --- | --- |
| 1 | bnlg2180 | 1 |
| 2 | phi094 | 1 |
| 3 | phi29852 | 1 |
| 4 | umc1254 | 1 |
| 5 | umc1353 | 1 |
| 6 | umc2028 | 1 |
| 7 | umc2096 | 1 |
| 8 | phi095 | 1 |
| 9 | umc1706 | 1 |
| 10 | bnlg1953 | 1 |
| 11 | bnlg1124 | 1 |
| 12 | bnlg2204 | 1 |
| 13 | umc1611 | 1 |
| 14 | umc1988 | 1 |
| 15 | bnlg1458 | 1 |
| 16 | umc1514 | 1 |
| 17 | umc1035 | 1 |
| 18 | umc1245 | 1 |
| 19 | umc1626 | 1 |
| 20 | umc2007 | 2 |
| 21 | umc1485 | 2 |
| 22 | bnlg1887 | 2 |
| 23 | umc2403 | 2 |
| 24 | bnlg1175 | 2 |
| 25 | umc2002 | 3 |
| 26 | bnlg1496 | 3 |
| 27 | umc1667 | 4 |
| 28 | bnlg1879 | 5 |
| 29 | bnlg1346 | 5 |
| 30 | umc1517 | 6 |
| 31 | bnlg2191 | 6 |
| 32 | bnlg1371 | 6 |
| 33 | mmc0523 | 6 |
| 34 | umc1006 | 6 |
| 35 | bnlg2132 | 7 |
| 36 | bnlg1131 | 8 |
| 37 | bnlg1194 | 8 |
| 38 | umc1828 | 8 |
| 39 | bnlg128 | 9 |
| 40 | umc1231 | 9 |
| 41 | bnlg1360 | 10 |
| 42 | umc1196 | 10 |
| 43 | umc1962 | 10 |

**Supplementary Table 2: Description and function of genes within *qLIR_9.1***

| **S No.** | **Gene Id** | **Description** |
| --- | --- | --- |
| 1 | MIR399j | zma-miR399j-3p |
| 2 | si605018e07b | WAT1 related protein |
| 3 | LOC100281455 | Ubiqutin-protein ligase |
| 4 | LOC103638121 | tRNA phosphotransferase |
| 5 | LOC109942267 | transcription factor |
| 6 | LOC100193354 | trafficking protein |
| 7 | LOC100144300 | TMV-MP 30 protein |
| 8 | LOC103639848 | testis specific gene |
| 9 | LOC103638162 | syntaxin-42 transcript variant |
| 10 | LOC103638113 | SWI- SNF 2 complex |
| 11 | LOC109942504 | sulfated surface glycoprotein |
| 12 | ss1 | starch synthase |
| 13 | LOC103638144 | SPX domain-containing membrane protein |
| 14 | LOC100282621 | splicing factor transcript variant |
| 15 | LOC103639903 | spidorin 1 |
| 16 | LOC103638287 | sister chromatid cohesin protein transcript variant |
| 17 | LOC100216656 | signal recognition particle |
| 18 | LOC103638146 | short chain dehydrogenase |
| 19 | LOC103644544 | serine threonine protein |
| 20 | LOC103638252 | senescence -specific cystine protease |
| 21 | LOC103638253 | senescence -specific cystine protease |
| 22 | LOC103639836 | selenium binding |
| 23 | ANAC076 | secondary wall NAC transcription factor |
| 24 | LOC100193146 | Sec14p-like transfer |
| 25 | LOC100273575 | Sec14p-like phosphatidylinositol like family |
| 26 | LOC100272800 | scarecrow-like |
| 27 | LOC100382171 | SAUR like auxin responsive protein family |
| 28 | LOC542185 | rubisco binding subunit large subunit binding%20protein |
| 29 | LOC103638047 | rRNA processing protein |
| 30 | LOC109942505 | RNA-binding%20protein%20cabeza binding |
| 31 | LOC103638069 | RING H finger protein |
| 32 | LOC100284868 | RING zinc finger protein like |
| 33 | LOC100284475 | ribonucleoside diphosphate reductase small chain |
| 34 | LOC103638184 | rho GTPase-activating activating protein REN 2C |
| 35 | rf2 | restorer of fertility protein |
| 36 | LOC103638036 | regulator nonsense transcripts homolog |
| 37 | pco146545 | Pyridoxal 27phosphate -dependent enzyme |
| 38 | LOC109942412 | protein trichome birefringence like |
| 39 | LOC103638218 | protein NSP INTERACTING KINASE |
| 40 | LOC100191990 | protein NRT1 family |
| 41 | LOC103638194 | protein ME12 like transcript variant |
| 42 | LOC103638128 | Protein EXORDIUM |
| 43 | LOC100282967 | protein binding protein transcript variant |
| 44 | LOC109942259 | protein ALP1 ALP |
| 45 | LOC109942259 | protein ALP1 ALP |
| 46 | pco130904 | Proteasome subunit beta type |
| 47 | LOC103638154 | Disease resistance |
| 48 | LOC103639806 | probable CCR 4 associated factor |
| 49 | LOC103638071 | pentatricopeptide protein containing variant |
| 50 | LOC100216695 | OSJNBa0089e12.13 like protein |
| 51 | LOC100282658 | nudix hydrolase |
| 52 | LOC103639772 | nuclear pore complex |
| 53 | znod1 | nodulation homolog |
| 54 | LOC111590117 | motile domain containing protein |
| 55 | LOC100383470 | Monocopper oxidase like protein |
| 56 | MIR399j | microRNA MIR399j |
| 57 | LOC100273442 | Lung seven transmembrane receptor family protein |
| 58 | LOC100501088 | Lipase-like transcript variant |
| 59 | LOC103638152 | LIMR family protein |
| 60 | kin2 | kinesin like protein |
| 61 | LOC100383377 | keratin associated protein |
| 62 | LOC103638266 | kanadaptin |
| 63 | pco131654 | IQ domain transcript variant |
| 64 | LOC103647554 | inosine triphosphate pyrophosphatase |
| 65 | LOC100381992 | hydrolase like protein |
| 66 | LOC100502355 | HXXXD type acyl transferase family protein |
| 67 | LOC103638100 | homeobox leucine zipper protein |
| 68 | LOC103638137 | Histone H3 |
| 69 | LOC103638044 | Histone H3 |
| 70 | LOC103638138 | Histone H3 |
| 71 | LOC103638138 | Histone H3 |
| 72 | LOC100281810 | hexose carrier protein |
| 73 | LOC100285600 | heat shock protein |
| 74 | LOC103638263 | hat transposon superfamily protein |
| 75 | LOC103639872 | golgin superfamily |
| 76 | LOC100502305 | glycine rich protein |
| 77 | LOC109942508 | glycine rich protein |
| 78 | LOC100273960 | GDSL esterase |
| 79 | LOC100281844 | formiminotransferase-like transcript |
| 80 | L-FNRI | ferroxidoxin |
| 81 | LOC103639850 | expansin A-17 |
| 82 | LOC542727 | enolpyruvylshikimate phosphate synthase |
| 83 | eno1 | enolase |
| 84 | LOC103638165 | endoplasmic reticulum metallopeptidase |
| 85 | LOC100273376 | Embryogeneis transmembrane |
| 86 | LOC103638160 | elongator complex protein |
| 87 | LOC109939243 | early nodulin |
| 88 | LOC103638099 | early nodulin |
| 89 | LOC103638198 | ubiquitin protein ligase RING like protein |
| 90 | d3 | dwarf plant transcript variant |
| 91 | LOC103639869 | dnaJ protein homolog |
| 92 | LOC103638270 | DNA topoisomerase transcript variant |
| 93 | LOC103638286 | DNA oxidative methylase |
| 94 | LOC103638180 | DExH-box RNA |
| 95 | LOC103638177 | DeSI-like protein |
| 96 | pco071268 | deoxy xylulose synthase |
| 97 | LOC100216961 | Dehydrin family protein expressed |
| 98 | LOC100283838 | DAG protein |
| 99 | LOC100502310 | cystine synthase transcript variant |
| 100 | conz1 | cystine synthase transcript variant |
| 101 | LOC109942478 | collagen alpha 1 chain like |
| 102 | LOC103638095 | coatmer subunit transcript variant |
| 103 | LOC103638293 | GRAM domain containing protein |
| 104 | LOC109942404 | BTB domain protein |
| 105 | LOC100272392 | binding |
| 106 | LOC103638278 | bifunctional phosphatase IMPL2 |
| 107 | LOC103638278 | bifunctional phosphatase IMPL2 |
| 108 | LOC103639852 | benzyl alcohol benzyltransferase |
| 109 | LOC103639852 | benzyl alcohol benzyltransferase |
| 110 | LOC100273963 | BBR FBPC transcription factor |
| 111 | LOC103638234 | basic blue protein |
| 112 | baf1 | barren stalk fastigiate |
| 113 | LOC109942360 | cystine synthase chaperone regulator |
| 114 | LOC109942396 | autophagy related protein |
| 115 | LOC103638045 | Atpob1 |
| 116 | LOC100283946 | ATP synthase |
| 117 | LOC103639824 | atherin-like |
| 118 | LOC103639824 | atherin-like |
| 119 | LOC100384150 | ARM repeat superfamily protein |
| 120 | LOC100502506 | Aluminium activated malate transporter |
| 121 | LOC109942480 | alanine and glycine rich protein like |
| 122 | LOC103638230 | acylphosphatase-1-like |
| 123 | LOC100280786 | acylphosphatase |
| 124 | TIDP3592 | Acyl-CoA-binding protein |
| 125 | LOC100273408 | AAA type ATPase family protein |
| 126 | LOC103638199 | 40S ribosomal protein S |
| 127 | LOC103638222 | 3 ketoacyl CoA synthase |
| 128 | LOC100281274 | 39S ribosomal protein transcript variant |
| 129 | LOC109942418 | N debenzoyl deoxytaxol N benzoyltransferase |
| 130 | LOC100279580 | 1-aminocyclopropane-1-carboxylate synthase3 |

**Supplementary Table 3: Description and function of genes within *qDH_2.1***

| **S No.** | **Gene Id** | **Description** |
| --- | --- | --- |
| 1 | GRMZM2G044800 | 40S ribosomal protein S11 |
| 2 | GRMZM2G010257 | Acidic ribosomal protein P2a-3 |
| 3 | GRMZM2G010257 | Acidic ribosomal protein P2a-3 |
| 4 | GRMZM2G173341 | Alpha-1,4-glucan-protein synthase |
| 5 | GRMZM2G102349 | Alternative oxidase |
| 6 | GRMZM2G010555 | Alternative oxidase |
| 7 | GRMZM2G076826 | APO protein 4 mitochondrial |
| 8 | GRMZM2G156581 | APO protein 4 mitochondrial |
| 9 | GRMZM2G093632 | Carbohydrate-binding X8 domain superfamily protein |
| 10 | GRMZM2G032209 | CDT1a protein |
| 11 | GRMZM2G086801 | COP9 signalosome complex subunit 5b |
| 12 | GRMZM2G040920 | Core-2 FI branching beta16- N acetylglucosaminyltransferase family protein |
| 13 | GRMZM2G064212 | Cysteine protease |
| 14 | GRMZM2G010435 | Cysteine-protease |
| 15 | GRMZM2G040736 | cytokinin response regulator |
| 16 | GRMZM2G106578 | Diacylglycerol kinase |
| 17 | GRMZM2G106578 | diacylglycerol-kinase |
| 18 | GRMZM2G005310 | DNA methyl transferase |
| 19 | GRMZM2G068967 | Ethylene-responsive transcription factor |
| 20 | GRMZM2G379758 | Eukaryotic translation initiation factor |
| 21 | GRMZM2G102069 | evolutionarily conserved terminal region |
| 22 | GRMZM2G009413 | Exosome complex RRP4 like protein |
| 23 | GRMZM2G022192 | Glutamyl-tRNA amidotransferase |
| 24 | GRMZM2G022192 | Glutamyl-tRNA amidotransferase |
| 25 | GRMZM2G023163 | Glycerol kinase |
| 26 | GRMZM2G160585 | Glycosyltransferases |
| 27 | GRMZM2G106245 | GNAT transcription factor |
| 28 | GRMZM2G121063 | High-affinity potassium transporter |
| 29 | GRMZM2G001289 | Homeodomain leucine zipper family |
| 30 | GRMZM2G076239 | Hydroxyacid oxidase |
| 31 | GRMZM2G094255 | Imidazoleglycerol-phosphate dehydratase |
| 32 | GRMZM2G040095 | Lipoxygenase |
| 33 | GRMZM2G156356 | Maltose excess protein |
| 34 | GRMZM2G302701 | Mannitol dehydrogenase |
| 35 | GRMZM2G130889 | Mannosyl-oligosaccharide |
| 36 | GRMZM2G477063 | Methionyl-tRNA |
| 37 | GRMZM2G084938 | N-acetylglucosaminylphosphatidylinositol protein |
| 38 | GRMZM2G086489 | NADPH--cytochrome reductase |
| 39 | GRMZM2G032977 | Nuclease PA |
| 40 | GRMZM2G456132 | Nucleic acid binding protein |
| 41 | GRMZM2G040878 | PAP fibrillin family |
| 42 | GRMZM2G156365 | Pectinacetylesterase |
| 43 | GRMZM2G352359 | Pectin esterase |
| 44 | GRMZM2G106384 | Pentatricopeptide repeat containing protein |
| 45 | GRMZM2G005107 | Peroxisomal C dienoyl- CoA reductase |
| 46 | GRMZM2G164400 | Peroxisome biogenesis protein |
| 47 | GRMZM2G165535 | Phosphomannomutase |
| 48 | GRMZM2G165535 | Phosphomannomutase |
| 49 | GRMZM2G096546 | Plant-specific family protein |
| 50 | GRMZM2G019404 | Plasma membrane ATPase |
| 51 | GRMZM2G012088 | Polyadenylate-binding protein |
| 52 | GRMZM2G045596 | Polygalacturonase |
| 53 | GRMZM2G091302 | Polyketide synthesis homolog |
| 54 | GRMZM2G005040 | Probable potassium transporter |
| 55 | GRMZM2G104538 | Probable phytol kinase chloroplastic |
| 56 | GRMZM2G102088 | Protein kinase- superfamily protein |
| 57 | mis12-2 | Protein MIS12- like |
| 58 | GRMZM2G084958 | Protochlorophyllide-reductase |
| 59 | GRMZM2G042933 | Putative amino- acid permease |
| 60 | GRMZM2G174784 | Putative AP FEREBP transcription factor family |
| 61 | GRMZM2G007791 | Putative argonaute- family |
| 62 | GRMZM2G131961 | Putative bZIP transcription factor |
| 63 | GRMZM2G020843 | Putative homeodomain-responsive transcription factor superfamily protein |
| 64 | GRMZM2G040115 | Putative phototropic-responsive NPH3 family protein |
| 65 | GRMZM2G104538 | Putative phytol kinase, chloroplastic |
| 66 | GRMZM2G059740 | Putative protein kinase superfamily protein |
| 67 | GRMZM2G165060 | Putative protein kinase superfamily protein |
| 68 | GRMZM2G391741 | Putative receptor-like protein kinase |
| 69 | GRMZM2G455978 | Putrescine-binding periplasmic protein-related |
| 70 | GRMZM2G054225 | Required to maintain repression 7 |
| 71 | GRMZM2G457621 | Rubisco accumulation factor 1, chloroplastic |
| 72 | GRMZM2G004590 | Shikimate kinase 1 chloroplastic |
| 73 | GRMZM2G076539 | Signal recognition particle 14 kDa protein |
| 74 | GRMZM2G040513 | Small glutamine-rich tetratricopeptide repeat-containing protein 2 |
| 75 | GRMZM2G155877 | Tetraspanin-19 |
| 76 | GRMZM2G156227 | Thylakoid lumenal 29 kDa protein chloroplastic |
| 77 | GRMZM2G156227 | Thylakoid lumenal 29 kDa protein chloroplastic |
| 78 | GRMZM2G098214 | tRNA methyltransferase catalytic subunit TRM61 |
| 79 | GRMZM2G102346 | Trypsin family protein |
| 80 | GRMZM2G145816 | Ubiquitin carboxyl-terminal hydrolase 22 |
| 81 | GRMZM2G023239 | Ubiquitin carboxyl-terminal hydrolase 22 |
| 82 | GRMZM2G175362 | Ubiquitin-specific protease family C19-related protein |
| 83 | GRMZM2G044629 | UDP-N-acetylglucosamine diphosphorylase 2 |
| 84 | GRMZM2G040627 | Uracil-DNA glycosylase |
| 85 | GRMZM2G040627 | Uracil-DNA glycosylase |
| 86 | GRMZM2G392125 | Xyloglucan endotransglucosylase/hydrolase protein 15 |
